# Cold atmospheric plasma stabilizes mismatch repair for effective, uniform treatment of diverse colorectal cancer cell types

**DOI:** 10.1038/s41598-024-54020-0

**Published:** 2024-02-13

**Authors:** Yuanyuan He, Fu Lu, Chenmin Jiang, Fanwu Gong, Zhengwei Wu, Kostya Ostrikov

**Affiliations:** 1 School of Nuclear Science and Technology, University of Science and Technology of China, Hefei, 230026 China; 2 Department of Geriatrics, The First Affiliated Hospital of USTC, Division of Life Sciences and Medicine, University of Science and Technology of China, Hefei, 230001 China; 3 School of Pharmacy, Anhui Medical University, Hefei, 230032 Anhui China; 4 Department of Medical Oncology, The First Affiliated Hospital of USTC, Division of Life Sciences and Medicine, University of Science and Technology of China, Hefei, 230001 China; 5 School of Chemistry and Physics and QUT Centre for Biomedical Technologies, Queensland University of Technology (QUT), Brisbane, QLD 4000 Australia

**Keywords:** Medical research, Colorectal cancer, Cancer, Plasma physics

## Abstract

Mismatch Repair (MMR) mechanisms play a pivotal role in rectifying DNA replication errors and maintaining the stability of DNA microsatellite structure. Colorectal cancer (CRC) can be characterized into microsatellite stability (MSS) and microsatellite instability (MSI) subtypes based on the functionality of MMR. MSI CRC notably exhibits enhanced chemotherapy resistance, attributable to diminished MMR-related protein expression. Cold atmospheric plasma (CAP) has emerged as a promising treatment modality, demonstrating efficacy in inducing apoptosis in various cancer cells. However, the therapeutic impact of CAP on MSI colorectal cancer, and the underlying mechanisms remain elusive. In this study, we investigated the effects of CAP on MSI (MC38, HCT116, and LOVO) and MSS (CT26 and HT29) CRC cell lines. We are probing into the products of CAP treatment. Our findings indicate that CAP treatment induces comparable effects on apoptosis, reactive oxygen species (ROS), and reactive nitrogen species (RNS), as well as the expression of apoptosis-related proteins in both MSI and MSS cells. Mechanistically, CAP treatment led to an elevation in the expression of mismatch repair proteins (MLH1 and MSH2), particularly in MSI cells, which notably have been proven to facilitate the activation of apoptosis-related proteins. Collectively, our study reveals that CAP enhances apoptotic signaling and induces apoptosis in MSI colorectal cancer cells by upregulating the expression of MMR-related proteins, thereby reinforcing MMR stabilization.

## Introduction

Colorectal cancer(CRC) is a prevalent malignancy, ranking third globally in terms of morbidity^1^. Despite the availability of various treatment modalities such as surgery, radiotherapy, chemotherapy, molecular-targeted therapy, and immunotherapy, their effectiveness is often hindered by intricate gene-regulated protein expression in cancer cells. The mismatch repair (MMR) mechanism, responsible for correcting errors in DNA replication, plays a crucial role in developing colorectal cancer (CRC). Notably, MMR function-deficient cancer cells exhibit microsatellite instability(MSI), characterized by DNA replication errors and misrepair^2^. These mutations impact a range of genes, including tumor suppressor genes and DNA repair genes. Mutations in these genes can result in uncontrolled cell growth and hinder differentiation, ultimately contributing to cancer development^3^. Colorectal cancer cells featuring abnormal DNA Mismatch Repair (MMR) protein function exhibit a mismatch repair deficiency (dMMR) / microsatellite instability (MSI) phenotype which confers them more resistant to chemotherapeutic agents compared to colorectal cancer cells with proficient mismatch repair (pMMR) / microsatellite stability (MSS)^2,3^. MSI colorectal cancer cells, marked by high heterogeneity, defective DNA MMR mechanisms, resistance to multiple chemotherapeutic agents, low survival rates, elevated metastasis, and poor treatment outcomes, pose significant therapeutic challenges^4,5^. The genetic and epigenetic heterogeneity inherent in these cells complicates the identification of common therapeutic targets. This article comprehensively addresses the intricate landscape of CRC, illuminating the mechanisms of MMR, the challenges posed by MSI colorectal cancer, and potential avenues for therapeutic intervention. One possible therapeutic strategy involves transforming MSI colorectal cancer cells into MSS status through epigenetic therapies. This could be accomplished using epigenetic therapies such as DNA methyltransferase or histone deacetylase inhibitors. However, the high heterogeneity of MSI colorectal cancer cells complicates the identification of suitable candidates for this approach. Additionally, managing CRC with an MSI phenotype, resistant to cytotoxic anticancer drugs, including oxaliplatin, irinotecan, and molecular-targeted therapies, presents a formidable challenge. Although immune checkpoint inhibitors have demonstrated safety and efficacy as first-line therapy for MSI-high colorectal cancer patients^6^, the overall recovery rates persist at a relatively modest level, and patients are susceptible to significant complications, notably immune-associated myocarditis^7^.

Therefore, there is a need for oncotherapy alternatives that are more adaptable and remain unaffected by gene expression. In this study, we identify a promising method in which Cold Atmospheric Plasma (CAP) demonstrates efficacy in treating MSI colorectal cancer, inducing apoptosis through increased expression of MMR-related proteins, thereby promoting the stabilization of the mismatch state.

CAP demonstrates extensive applicability in eliminating human cancer cells, supported by numerous in vivo and in vitro studies^8,9^. The primary anti-tumor mechanism of CAP involves generating ROS and RNS, leading to cell death^10^, inhibiting cell proliferation^11^, inducing DNA damage^12^, and activating signaling pathways^13^ in cancer cells. CAP is significantly superior in the induction of apoptosis and necrosis in a variety of malignant tumor cells, including both MSI and MSS phenotypes, as observed in melanoma cells (MSI)^14^, breast cancer cells (MSI)^15^, oral cancer cells (MSI)^16^ colorectal cancer cells (MSI)^17,18^, cervical cancer cells (MSI)^19^, lung cancer cells (MSI)^20,21^, bladder cancer cells (MSI)^22^, prostate cancer cells (MSS)^23^ and pancreatic cancer cells (MSS)^24^. Overall, these findings emphasize the substantial potential of CAP as an onco-therapeutic tool, particularly in contrast to conventional chemotherapy, for MSI tumor cells with MMR deficiency. However, limited research has compared the therapeutic effects of CAP on tumor cells in microsatellite-stable and microsatellite-unstable conditions. Further investigations are needed to explore the underlying mechanisms. In the present study, we aimed to investigate CAP's effects and underlying mechanisms on colorectal cancer cells with MSI statuses, explicitly focusing on the correlation between ROS/RNS levels and cell apoptosis. Our findings reveal that CAP treatment induces necrosis and apoptosis in MSI and MSS colorectal cancer cells by increasing intracellular ROS/RNS levels and enhancing pro-apoptotic protein expression. Particularly noteworthy is the observed upregulation of MLH1 and MSH2 in MSI cells. Furthermore, our research outcomes indicate a significant potential of Cold Atmospheric Plasma (CAP) treatment in vitro to induce the transformation of Microsatellite Instability (MSI) colorectal cancer cells into the Microsatellite Stable (MSS) phenotype. Consequently, we propose that CAP therapy exhibits the dual capacity to independently eliminate colorectal cancer cells and serve as a supplementary approach to restore the sensitivity of MSI colorectal cancer cells to chemotherapeutic agents.

## Results

**Figure 1:**
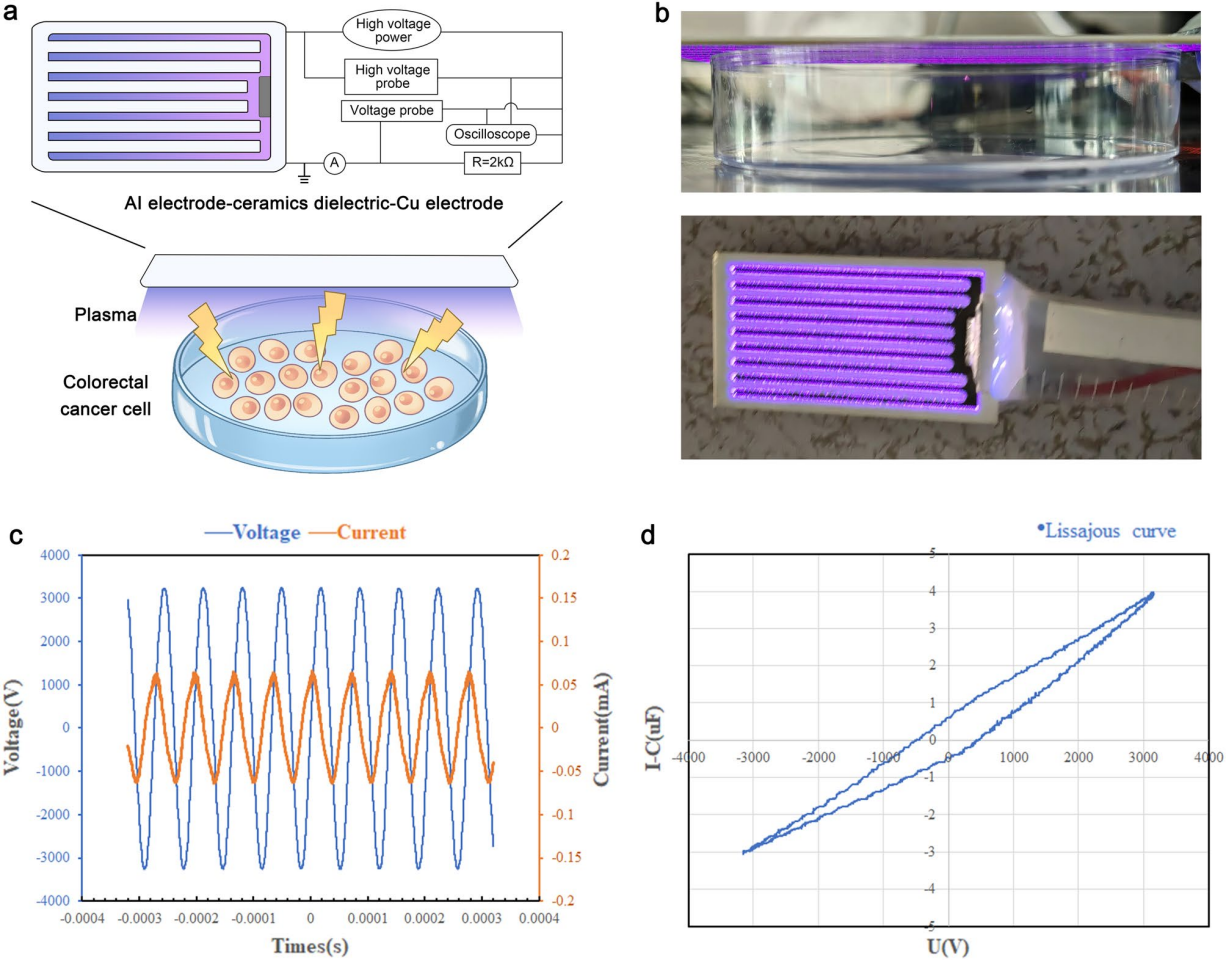
Schematics of the experimental Dielectric Barrier Discharge (DBD) plasma device for Colorectal cancer cell treatment. (**a**) The apparatus comprises an Al electrode-ceramics dielectric-Cu electrode, an AC power supply, an oscilloscope, a high-voltage probe, a microammeter, a low-voltage probe, and a resistor. (**b**) The plasma is generated from the DBD plasma device's electrode onto the culture medium's surface. (**c**) The voltage and current waveforms of the DBD plasma device discharge. (**d**) The Lissajous figure of the DBD discharge.

The impact of CAP therapy on colorectal cancer cells in vitro were systematically investigated using the Dielectric Barrier Discharge (DBD) plasma device. A detailed explanation of the DBD plasma device and treatment parameters is provided in Fig. 1—the experimental procedures involved applying 60,120 and 180 s of DBD plasma treatment for in vitro cell cultures.

### CAP treatment enhances cytotoxicity and Induces apoptosis in colorectal cancer cells independent of microsatellite stability phenotype

**Figure 2:**
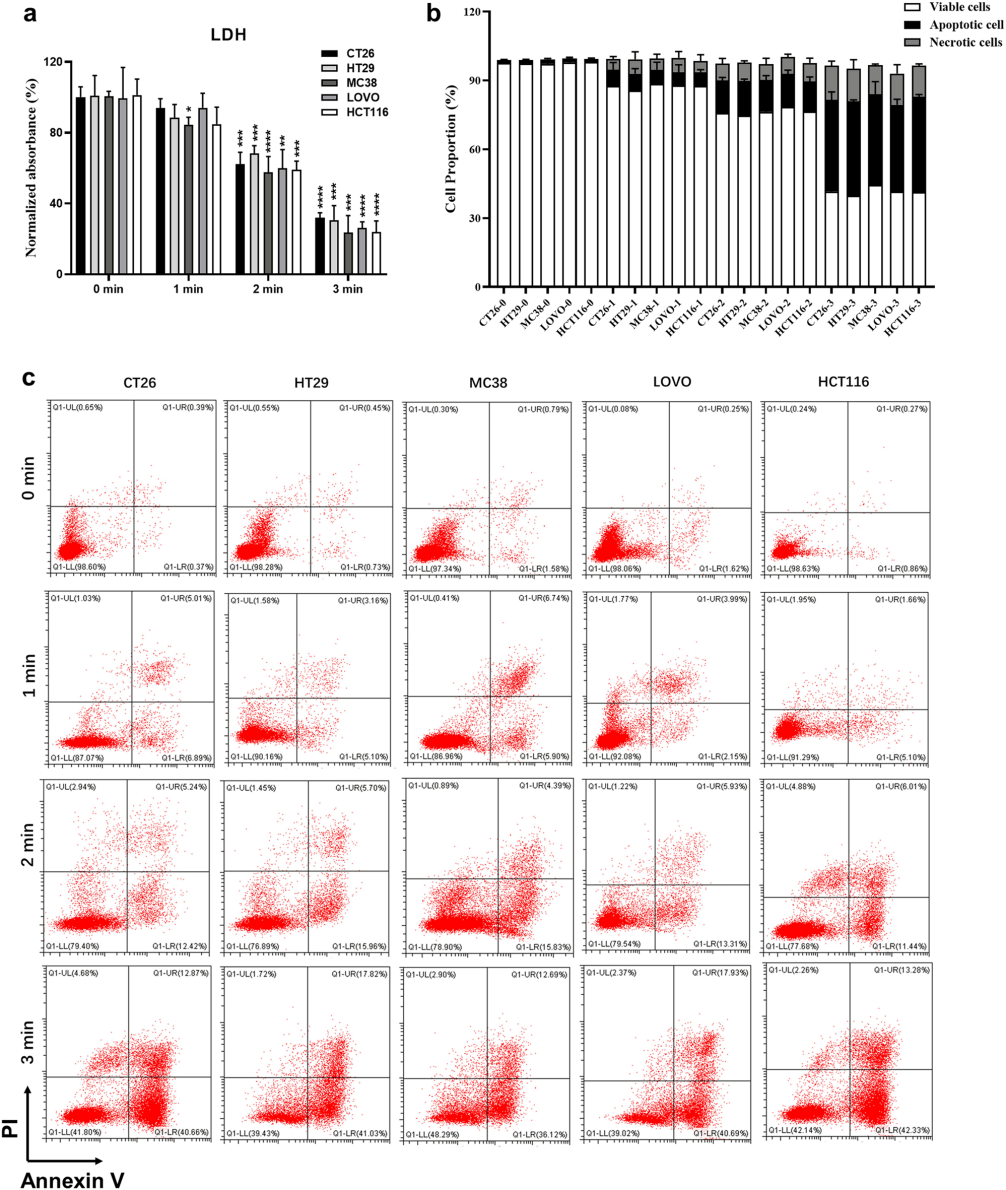
CAP treatment-induced cytotoxicity and apoptosis in HCT116, LOVO, MC38, HT29, and CT26 cells in a time-dependent manner. Colorectal cancer cells are cultured for three days. Three different exposure times of CAP treatment were investigated. (**a**) Normalized absorbance values of LDH (OD490) assays were conducted with five cell lines in both the control and CAP treatment groups. n = 4. (**b**, **c**) Flow cytometry analyses based on Annexin-V/PI staining to determine apoptotic cells in all groups (control, CAP treatment for 60, 120, and 180 s), with percentages of apoptotic cells (**b**) and logarithmic plots (**c**), n = 3. The data represent at least three independent experiments (means ± SD). The significance of the CAP treatment groups (1 min, 2 min, and 3 min) was determined using two-sided unpaired t-tests compared to the control groups (0 min), respectively. (*p < 0.05, **p < 0.01, ***p < 0.001, and ****p < 0.0001). One-way ANOVA determined statistical analyses between the same treatment time groups.

The sensitivity of various colorectal cancer cell phenotypes to CAP treatment was systematically examined. The diverse colorectal cancer cells under investigation included cell lines with MSS characteristics (CT26 and HT29) and those characterized by MSI cell lines (MC38, HCT116, and LOVO). Assessments of intracellular levels of LDH and the proportion of apoptotic cells were analyzed 24 h after CAP treatment for 60, 120 and 180 s. The outcomes were compared to those of the untreated control group (Fig. 2). Intracellular LDH levels represent cytotoxicity and inversely correlate with cell survival (Fig. 2a).

Notably, the results showed that all cell lines exhibited a significant increase in cytotoxicity and apoptosis rates with extended CAP treatment durations compared to the control group. Furthermore, there was no statistically significant difference in survival and apoptosis rates between MSS colorectal cancer cells (HT29 and CT26 cells) and MSI cells (MC38, HCT116, and LOVO cells) after DBD treatment (Fig. 2b). These results indicate that CAP's induction of apoptosis in colorectal cancer cells remains unaffected by the cell's MMR phenotype.

### CAP treatment increases intracellular/extracellular ROS/RNS levels and induces cell membrane damage in colorectal cancer cells

**Figure 3:**
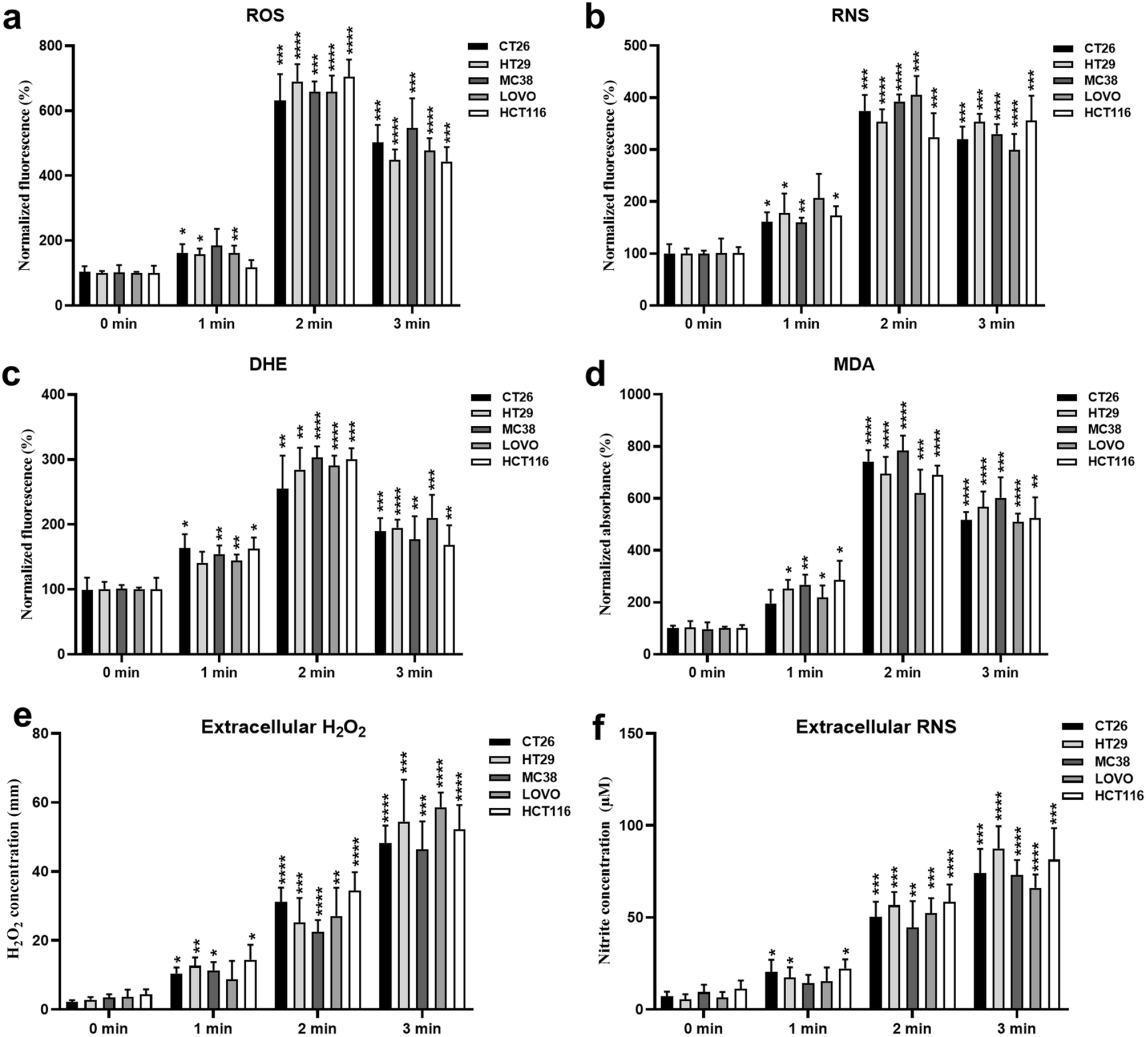
CAP treatment induced the generation of intracellular/extracellular ROS/RNS. (**a**, **b**, **c**) Intracellular ROS, RNS and superoxide anion (DHE, reflecting the level of intracellular oxidative stress) levels of different tumor cells treated with a time gradient of CAP, n = 3. (**d**) Membrane oxidation (MDA, reflecting the extent of oxidative damage to the cell membrane) levels of different tumor cells treated by time gradient of CAP, n = 3. The concentrations of H_2_O_2_ and nitrite in the supernatants of tumor cell cultures treated with a time gradient of CAP were measured to assess extracellular ROS (**e**) and RNS (**f**) levels, n = 6. The significance of the CAP treatment groups (1 min, 2 min, and 3 min) was determined using two-sided unpaired t-tests compared to the control groups (0 min), respectively. *p < 0.05, **p < 0.01, ***p < 0.001, and ****p < 0.0001). One-way ANOVA deter-mined statistical analyses between the same treatment time groups.

To elucidate the mechanism underlying the increased apoptosis of HCT116, LOVO, MC38, HT29, and CT26 cells upon CAP treatment, we applied the DBD CAP device to the aforementioned cells. Reactive oxygen species (ROS,·O^2-^) and reactive nitrogen species (nitric oxide NO) levels in colorectal cancer cells were detected as intracellular ROS/RNS at different plasma treatment times: 60, 120 and 180 s. We conducted intracellular total reactive oxygen species (ROS) detection (Fig. 3a), which includes the superoxide anion (O^2−^), hydrogen peroxide (H_2_O_2_), hydroxyl radical (·OH), and peroxides (ROO·). Subsequently, we specifically quantified the concentration of the intracellular superoxide anion (O^2−^) by measuring the intensity of the fluorescent signal of dioxyethidium (DHE) through a multi-mode microplate reader, thereby indirectly reflecting the level of intracellular oxidative stress (Fig. 3c). Nitric oxide (NO) stands for Reactive nitrogen species (Fig. 3b). Additionally, reactive oxygen species (H_2_O_2_) and total Nitric Oxide in tumor cell culture supernatants were detected as extracellular ROS/RNS at the same plasma treatment times (Fig. 3e,f). Malondialdehyde (MDA), the end product of membrane lipid peroxidation, was used to indirectly reflect the extent of oxidative damage to the cell membrane (Fig. 3d). As expected, CAP treatment elevated intracellular/extracellular ROS/RNS levels and increased the degree of cell membrane damage in a time-dependent manner. Apoptosis ratios were positively correlated with intracellular/extracellular reactive oxygen and nitrogen species (RONS) contents.

### CAP-derived H_2_O_2_ and NO lead to colorectal cancer cells damage and apoptosis

**Figure 4:**
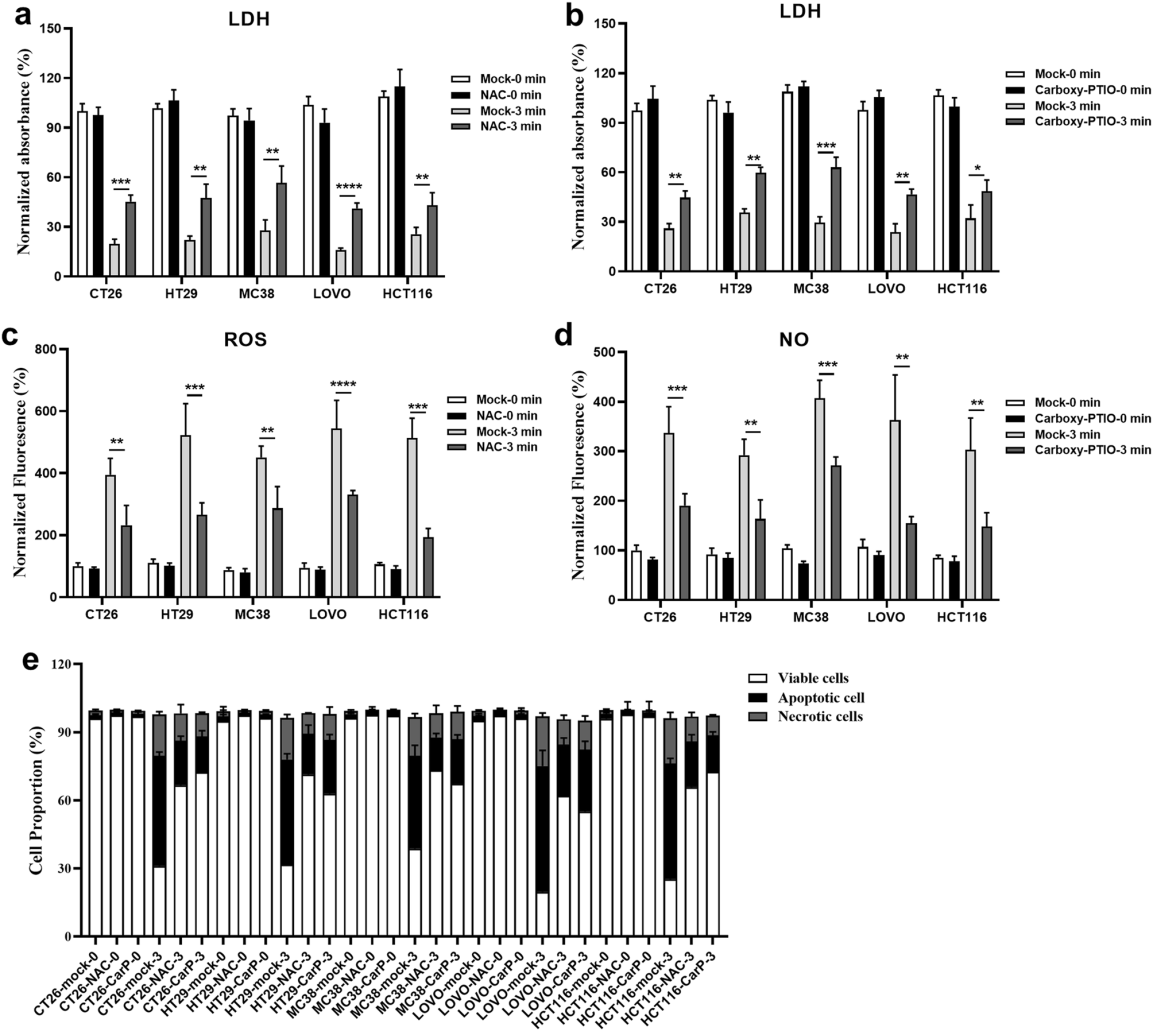
N-acetyl-L-cysteine (NAC) and Carboxy-PTIO inhibited CAP-induced damage and apoptosis in colorectal cancer cells. (**a**, **b**) The normalized absorbance values of LDH (OD490) assays were conducted with five cell lines in both pretreatment with RONS scavengers and non-pretreatment with RONS scavenger groups. Colorectal cancer cells were pretreated with 5 mM N-acetyl-L-cysteine (**a**) or 50 μM Carboxy-PTIO, CarP for short (**b**) for 2 h and then treated with CAP, n = 6. (**c**) The intracellular ROS of colorectal cancer cells treated with NAC described in (**a**), n = 6. (**d**) The intracellular NO of colorectal cancer cells treated with Carboxy-PTIO described in (**b**), n = 6. (**e**) Flow cytometry analyses based on Annexin-V/PI staining to determine percentages of apoptotic cells in all groups (control, NAC pretreatment and Carboxy-PTIO pretreatment), n = 6. The significance was determined using two-sided unpaired t-tests. *p < 0.05, **p < 0.01, ***p < 0.001, and ****p < 0.0001).

As an ionized gas, plasma comprises charged particles, radicals, and neutral molecules, showcasing diverse physical and chemical properties. In the context of oncology, outcomes are shaped by the involvement of reactive oxygen species (ROS), reactive nitrogen species (RNS), as well as charged and neutral particles, coupled with radiation from plasmas, resulting in the targeted elimination of diverse cancer cells^25–27^. While ROS and RNS play a significant role in inducing cellular responses to cold atmospheric plasma (CAP) treatment in both in vitro and in vivo settings, physical factors are not considered primary contributors to these effects^28–30^. To confirm the hypothesis that the antiproliferative activity of CAP on colorectal cancer cells is attributed to the release of ROS and RNS, we conducted tests in the presence or absence of the ROS scavenger, N-acetyl-L-cysteine (NAC), and the NO scavenger, Carboxy-PTIO. The principle of NAC antioxidant action is to enhance cellular antioxidant capacity by increasing intracellular glutathione content. This process promotes intracellular reduction reactions, subsequently reducing the generation and accumulation of reactive oxygen species. On the other hand, Carboxy-PTIO is a water-soluble and stable free radical that reacts stoichiometrically with NO, forming carboxy-PTI derivatives and generating nitrite or nitrate. This reaction aids in scavenging intracellular NO. Notably, NAC primarily scavenges the ROS component of cells, specifically H_2_O_2_, while carboxy-PTIO specifically targets the cellular NO component. The results were presented as the percentage of cell growth inhibition and apoptosis ratio in five colorectal cancer cell groups (Fig. 4). Intracellular levels of ROS and NO were measured in both NAC- and Carboxy-PTIO- treated and untreated groups. It was observed that both ROS (Fig. 4c) and NO (Fig. 4d) decreased after RONS scavenger treatments. Furthermore, CAP treatment for 180 s significantly inhibited cell growth and increased apoptosis in colorectal cancer cells. The inhibitory effects of cell growth (Fig. 4a,b) and apoptosis (Fig. 4e) were diminished by pretreatment with NAC and Carboxy-PTIO in a dose-dependent manner. These results demonstrated that H_2_O_2_ and NO produced by CAP treatment substantially contribute to inhibiting colorectal cancer cell proliferation in vitro.

### CAP treatment generated RON/RNS induced apoptosis in colorectal cancer cells by regulating apoptotic/anti-apoptotic proteins

**Figure 5:**
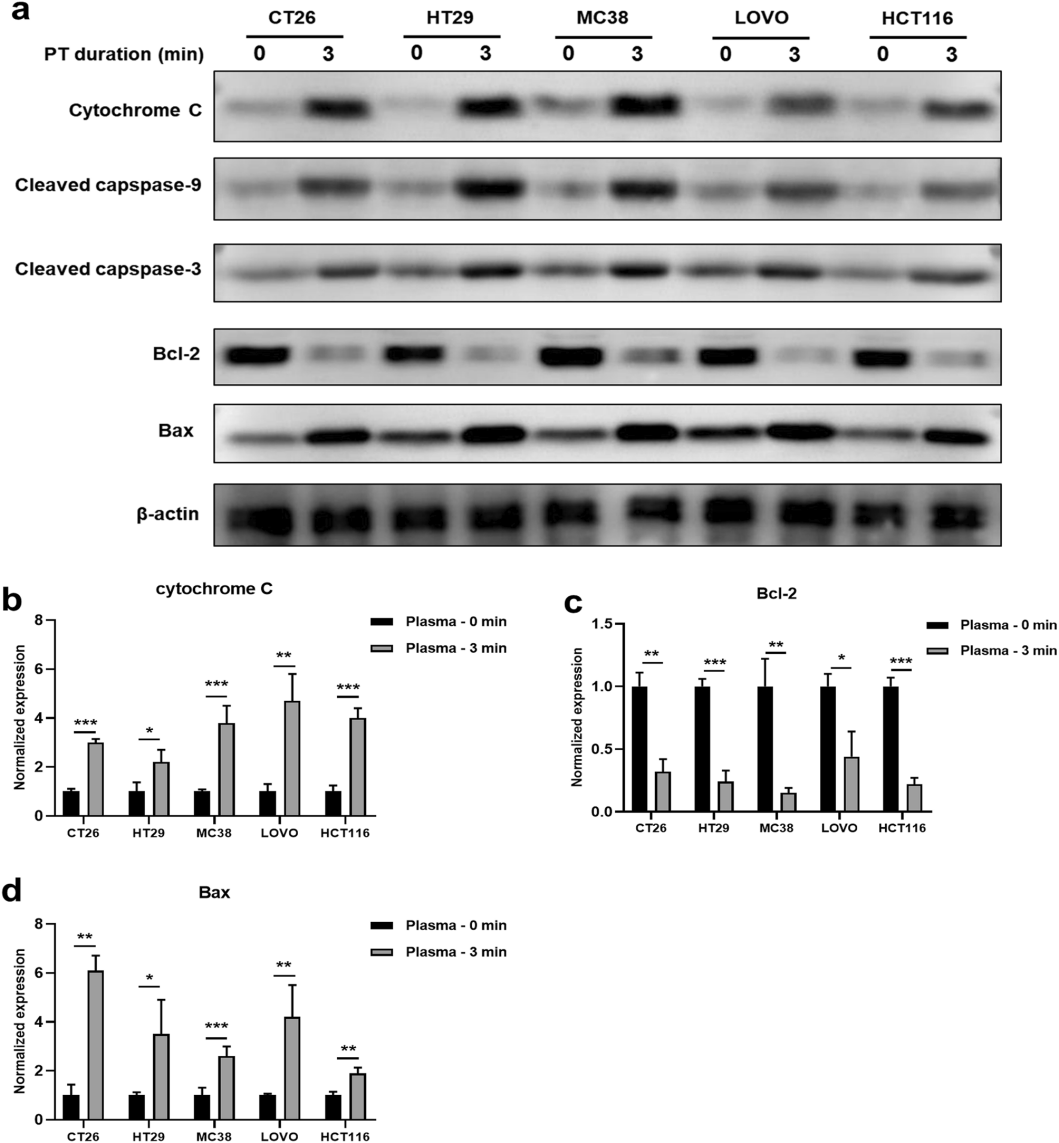
CAP treatment induced apoptosis in colorectal cancer cells by regulating apoptotic/anti-apoptotic proteins. (**a**) Cyt C, Cleaved caspase-9, Cleaved caspase-3, Bcl-2, and Bax proteins in indicated tumor cells treated by CAP were analyzed by immunoblotting. RT-qPCR analysis to determine the expressions of Cyt C (**b**), Bcl-2 (**c**), and Bax (**d**) in indicated tumor cells treated by DBD plasma. Statistical analyses between the 0 min control and 3 min DBD plasma treatment groups were determined by two-sided unpaired t-tests, respectively. (*p < 0.05, **p < 0.01 and ***p < 0.001).

Cytochrome-c (Cyt C) stands out as a critical player in the apoptotic cascade within mitochondria. Caspase-9, is a pivotal enzyme initiating the mitochondrial apoptotic signaling pathway^31,32^. Cyt C release triggers apoptosis by binding to apoptotic protease-activating factor 1(Apaf-1), inducing apoptosome assembly. Within the apoptosome, pro-caspase-9 undergoes self-activation, becoming an active enzyme. Active caspase-9 then initiates a cascade, activating effector caspases like caspase-3. Effector caspases execute apoptosis, leading to characteristic cellular changes^33,34^. To investigate whether CAP treatment induces apoptosis through the mitochondrial pathway, we conducted a Western blot analysis of apoptosis and anti-apoptosis-related proteins with varying expressions in HCT116, LOVO, MC38, HT29, and CT26 cells subjected to different durations of CAP treatment (Fig. 5). Firstly, CAP treatment significantly elevated the protein expression level of cytosolic cytochrome-c compared to the control groups (Fig. 5a). To further address the apoptotic effect of CAP on various colorectal cancer cells, we analyzed cleaved caspase-9 and cleaved caspase-3 levels. Following 3 min of CAP treatment, a substantial increase in cleaved caspase-9 and cleaved caspase-3 production was observed in CT26, HT29, MC38, LOVO and HCT116 cells. Next, in our exploration of the molecular events involved in CAP-induced apoptosis, we examined the expression of Bcl-2 and Bax, critical regulators of antioxidant and apoptosis pathways. The results demonstrated a decrease in the expression of the anti-apoptotic protein Bcl-2 in the CAP-treated groups. In contrast, the proapoptotic protein Bax increased in all five types of cancer cells accordingly (Fig. 5a). Results from RT-qPCR for Cyt C, Bcl-2, and Bax were consistent with these findings (Fig. 5b–d). These results indicate that CAP upregulates apoptotic signaling in Microsatellite Instability (MSI) and Microsatellite Stability (MSS) colorectal cancer cells.

### Plasma treatment affects MMR-related proteins expression in colorectal cancer cells dependent on the MSI status of the tumor cells

**Figure 6:**
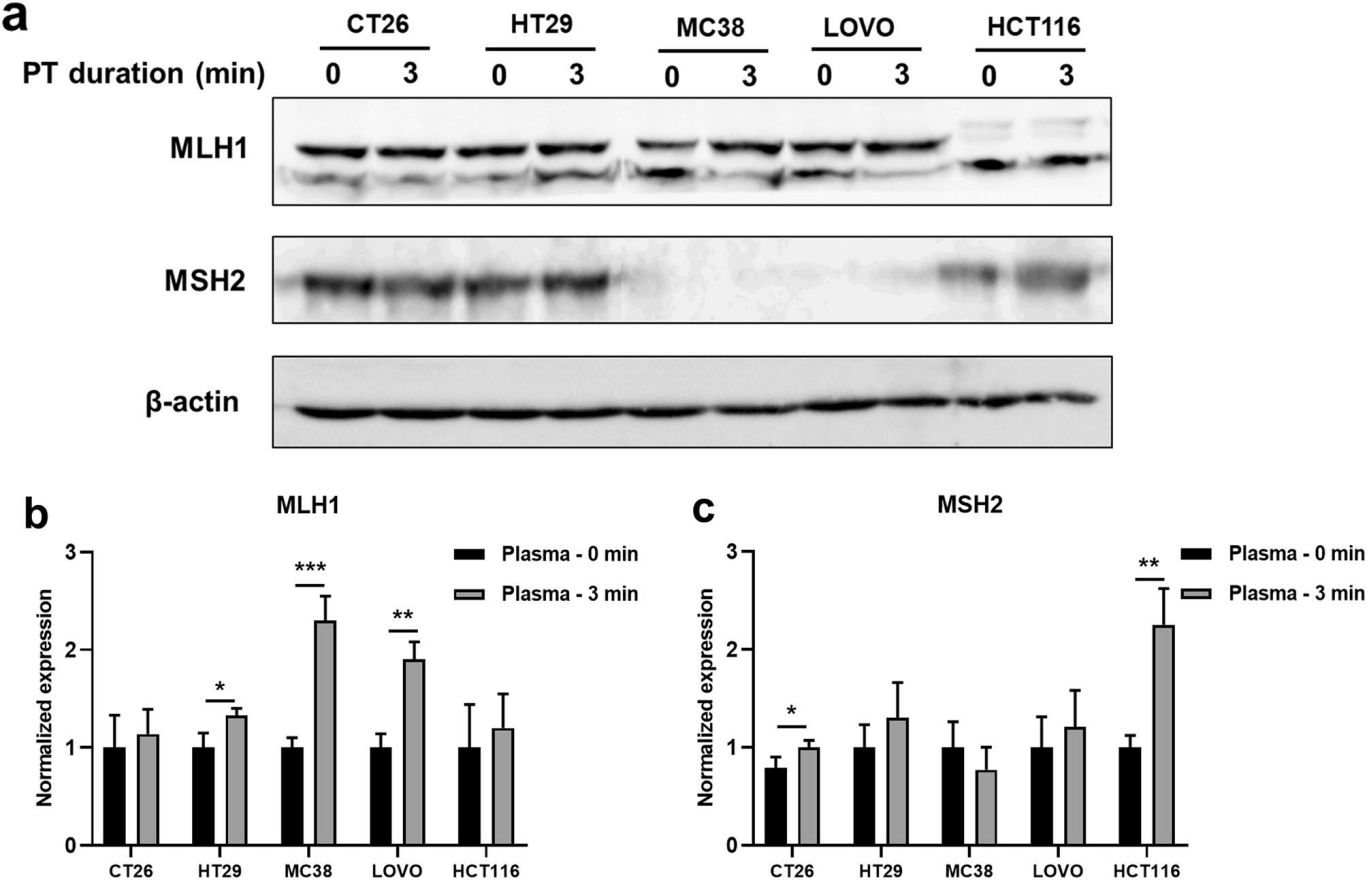
CAP treatment affects MLH1 and MSH2 expression in colorectal cancer cells dependent on the MSI status of the tumor cells. (**a**) MLH1 and MSH2 proteins in indicated tumor cells treated by CAP were analyzed by immunoblotting. Expression levels of MLH1 (**b**) and MSH2 (**c**) in indicated tumor cells treated by DBD plasma were analyzed by RT-qPCR. Statistical analyses between the 0 min control and 3 min DBD plasma treatment groups were determined by two-sided unpaired t-tests, respectively. ((*p < 0.05, **p < 0.01 and ***p < 0.001).

Most mutations contributing to the MSI state in cells occur in the MMR system, mainly affecting DNA MMR-related proteins like MLH1 and MSH2^35^. A search for MLH1 and MSH2 gene information in colorectal cells through the NCBI website revealed that HT29 and CT26 (MLH1 and MSH2 wild type) are MMR proficient cell lines. In contrast, MC38, LOVO (MSH2 methylated or mutated respectively, and MLH1 wild type) and HCT116 (MLH1 methylated or mutated respectively and MSH2 wild type) are MMR-deficient cell lines^36^. Immunoblotting analysis of MSI cells indicated that CAP treatment resulted in a significant increase in MLH1 expression in the MC38 and LOVO cell lines and a significant increase in MSH2 expression in the HCT116 cell line (Fig. 6a). In contrast, no obvious indication of MSH2 was observed in MSH2 protein methylated or mutated cell lines MC38, and LOVO, and no apparent MLH1 expression levels were observed in MLH1 protein methylated or mutated cell lines such as HCT116(Fig. 6a). Furthermore, the expression levels of MLH1 and MSH2 in the indicated tumor cells were analyzed by RT-qPCR, and the results were consistent with the western blotting results above (Fig. 6b,c). MLH1 and MSH2 levels also slightly increased in HT29 and CT26, rich in MLH1 and MSH2 protein expression (Fig. 6b,c). Together, these results suggest that CAP treatment can facilitate the presentation of DNA error-correcting proteins of MMR-deficient cells, maintaining DNA repair regulation and potentially promoting the reversal of tumor cells to MMR proficient status.

## Discussion

CAP-induced apoptosis in colorectal cancer cells is not dependent on the steady state of the cellular microsatellite. The MSI state, characterized by defects in the expression of MMR-related proteins such as MLH1 and MSH2 is a significant factor contributing to the insensitivity of colorectal cancer to chemotherapeutic drugs^37^. CAP releases various active ROS and RNS^38^, resulting in cell membrane damage^39^, cell cycle arrest^40^, and eventual cell necrosis and apoptosis^41,42^ with minimal damage to adjacent normal cells^43^. Notably, CAP has been shown to enhance the sensitivity of tumor cells to chemotherapeutics^44,45^.

Our study was designed to analyze the effects of plasma on colorectal cancer cells using DBD technology for plasma production in the air. First, we investigated whether similar results could be obtained in the molecular and cellular behavior of five colorectal cancer cells treated. Secondly, we aimed to determine if apoptosis rates correlate with intracellular RONS levels. Finally, we were interested in whether colorectal cancer cells with an MSI status react differently to CAP treatment. These findings offer crucial insights into the therapeutic response to CAP treatment.

In our study, we observed a distinct inhibition of cell proliferation in tumor cells after DBD treatment. Dielectric barrier discharge (DBD) plasma treatment for 60, 120, and 180 s significantly induced necrosis, apoptosis, and lipid peroxidation of the cell membrane in all five types of colorectal cancer cells. We achieved a consistent killing effect using CAP for different cell types. Meanwhile, CAP treatment was positively correlated with the levels of intracellular ROS and RNS, significantly down-regulated the ratio of anti-apoptotic protein Bcl-2 to apoptosis-associated protein Bax, and up-regulated the levels of cleaved caspase-3 and caspase-9. Recent studies have revealed that ROS/RNS can impose various biological effects on tumor cells depending on their concentration. Low concentrations of ROS/RNS play a role in the proliferation and differentiation of cells^46^. Simultaneously, when specific threshold densities of ROS/RNS are exceeded, apoptosis in tumor cells is induced^47^. CAP treatment may generate various ROS/RNS, inducing tumor cell apoptosis^18^. These findings showed a dramatic rise in ROS/RNS in a CAP treatment time-dependent manner, suggesting that ROS/RNS generation plays a critical role in the CAP-induced apoptosis in different types of colorectal cancer cells (HT29, CT26, LOVO, MC38, and HCT116) independently of their MSI status. This supports the idea that CAP treatment may be an effective therapy for colorectal cancer patients regardless of their specific cancer subtype.

After CAP treatment, significant changes were observed in MSI and MSS colorectal cancer cells regarding the expression of apoptosis and anti-apoptosis-related proteins, specifically Bax and Bcl-2. The inhibition of tumor cell proliferation and induction of tumor cell apoptosis are major strategies in tumor therapy, with the mitochondrial pathway being one of the most essential cellular apoptosis signal transduction pathways. Apoptosis, is regulated by Bax and Bcl-2 proteins, which are vital regulatory factors of this pathway. The physiological function of Bcl-2 is to prevent apoptosis and extend cell life without affecting the cell cycle and differentiation^48^. Conversely, Bax is a pro-apoptotic factor, and the intracellular expression of Bcl-2 and Bax remains relatively stable under normal conditions. When Bax is overexpressed intracellularly, the number of Bax/Bax homodimers significantly increases. Leading to the release of Cyt C from mitochondria into the cytoplasm. This activation of Caspase-3 initiates apoptosis^49^. Conversely, when Bcl-2 is highly expressed, the Bax/Bax dimer dissociates, exerting an anti-apoptotic effect that prolongs cell survival^50^. In the present study, Western blotting analysis suggested that both ROS and RNS generated by CAP could induce apoptosis in colorectal cancer cells by inhibiting Bcl-2 expression and promoting Bax, caspase-9, and caspase-3 expression at different treatment times^51^. This was further validated by the significant increase in apoptotic protein caspase-9 and caspase-3 expression, as detected by Western blotting. These results suggest that colorectal cancer cells are irreversibly inactivated by CAP treatment, regardless of their MSI status.

The primary function of the DNA MMR system is to repair mismatched bases during DNA replication, thereby maintaining genome stability. When MMR genes are mutated or methylated, it causes MMR-related proteins (such as MLH1, MLH3, MSH2, MSH3, MSH6, PMS1, and PMS2) to become mismatch repair deficient (MMR-deficient), This deficiency leads to the accumulation of mismatches in microsatellite DNA during replication, ultimately causing MSI^52^. Simultaneously, MSI patients show ineffectiveness to 5-FU chemotherapy^37^. It has been demonstrated that the promoter methylation of MMR-related proteins, particularly MLH1 or MSH2, primarily causes MSI. DNA methylation is one of the main factors contributing to MMR deficiency. ROS and RNS, crucial components of plasma, play a pivotal role in altering the levels of hypermethylated or hypomethylated genes in numerous cancer cells^9,53^.

Some studies have demonstrated that plasma can modulate epigenetic changes in cancer cells, resulting in altered gene expression and potentially sensitizing the cells to other cancer therapies^9^. Plasma has been shown to induce DNA methylation, leading to a low decrease in Alu sequence methylation in breast cancer MDA-MB-231 cell lines. Comprehensive DNA methylation profile changes were detected with microarray analysis, revealing 318 hypermethylated CpGs and 56 hypomethylated CpGs in breast cancer MCF-7 cells, as well as 76 hypermethylated CpGs and 63 hypomethylated CpGs in MDA-MB-231 cells^54^. Moreover, plasma can induce histone methylation, as evidenced by 899 sequences within promoter regions showing changes in H3K4me3 methylation levels after CAP application. Statistically significant correlations were observed between the expression of several genes and histone methylation changes^16^. Consequently, it is speculated that plasma-induced alterations in DNA and histone methylation may play a role in correcting promoter methylation. This correction could lead to increased expression levels of MMR-related proteins such as MLH1 or MSH2 in MSI colorectal cancer cells. Notably, MSS cells, lacking promoter mutations, exhibited no significant changes, as observed in HT29 and CT26 cells. Previous reports suggest that MMR-related proteins can recognize and recruit apoptosis-related molecules such as Cyt C in mitochondria. The release of Cyt C from mitochondria marks the initiation of apoptosis. Upon reaching the cytoplasm, Cyt C binds to Apaf-1, triggering a conformational change and the assembly of multiple Apaf-1 molecules into the apoptosome.^31,33,34^. This cascade eventually activates downstream apoptosis-related molecules, including caspase-9 and caspase-3, leading to cell death^32,55^. Further studies can be conducted by investigating the expression of methylated promoters.

The MSI status is a crucial factor contributing to insensitivity to various chemotherapeutic agents, including 5-FU, oxaliplatin, and irinotecan, in numerous tumors^37^. Our study reveals that plasma upregulated the expression of MMR-related proteins MLH1 and MSH2 in MSI status cells (MC38, HCT116, and LOVO cells), facilitating the transformation of MSI cells to MSS cell status. This observation holds significant relevance because MSI cells contribute to the chemoresistance observed in colorectal cancer cell lines such as MC38, LOVO, and HCT116 cells. Since our study confirms that the chemical reactivity of CAP is not constrained by the MSI status of colon tumor cells, the capability of CAP treatment to enhance chemosensitivity may enable the use of lower doses of chemotherapeutic agents. This can potentially reduce the risk of adverse effects while increasing treatment efficacy in the future^56^. Therefore, CAP is anticipated to play a critical role in reshaping the MSI status of tumor cells, mainly when used prior to chemotherapeutic treatments. This approach holds promise for enhancing the effectiveness of conventional treatments that have proven ineffective for MSI tumors.

**Figure 7:**
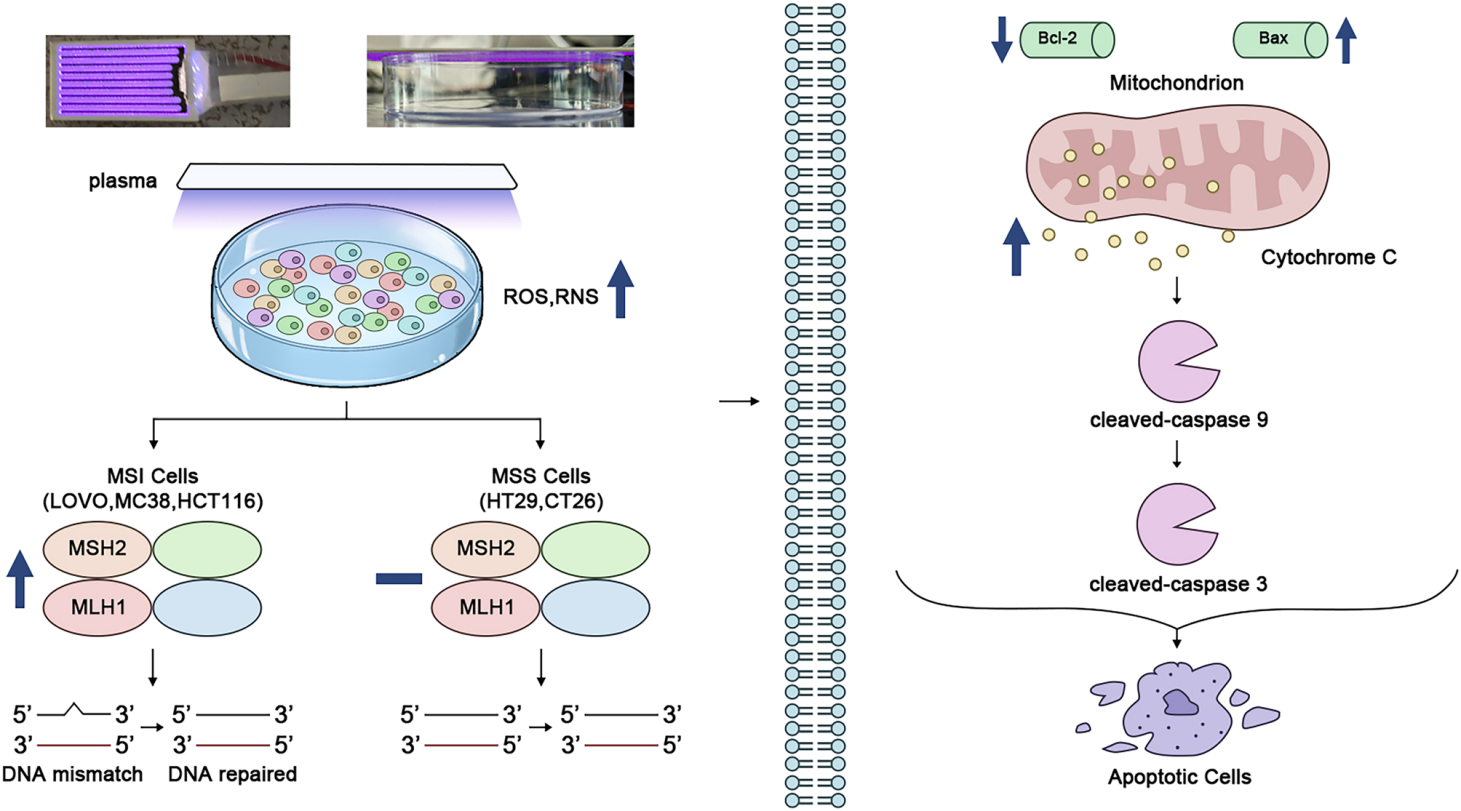
Proposed mechanism of CAP-induced cell death and cellular and stabilizing MMR in colorectal cancer cells. CAP, as a source of extracellular ROS, can elevate intracellular levels of ROS and RNS. High levels of ROS and RNS can upregulate the expression levels of MSH2 and MLH1 in MSI colorectal cancer cells (LOVO, MC38, HCT116), which can lead to the expression of MMR-related protein in MSI cells close to the expression levels in MSS colorectal cancer cells (CT26, HT29). Mediating DNA mismatch to DNA repaired in MSI and MSS colorectal cancer cells can induce apoptosis more efficiently and uniformly through the Cyt C-mediated Caspases 3/9 apoptosis pathway.

Our research indicates that CAP treatment effectively eliminates microsatellite unstable tumor cells by modulating the altered state of MMR-related proteins expression, as depicted in Fig. 7. This modulation leads to MMR stabilizing effectively and uniformly, addressing various types of colorectal cancer cells. The therapy can potentially enhance the antitumor response and regulate MMR function in MSI colorectal cancer. This discovery holds significant clinical implications, as CAP is a physical therapy drug utilized to treat multiple cancers. Our study presents a promising alternative strategy for addressing drug-resistant colorectal cancer cells, and it is anticipated to be employed in the future in combination with other conventional therapies to achieve improved results.

## Materials and methods

### Experimental setup

The low-temperature plasma device, utilizing Dielectric Barrier Discharge (DBD) technology, is detailed in Fig. 1. It consists of an Al electrode-ceramics dielectric-Cu electrode powered by a 220 V AC source (Fig. 1a). The Cu electrode, with a discharge area of 4.8 cm^2^, operates at atmospheric pressure using air as the working gas for plasma generation. The device discharge procedure is depicted in Fig. 1b. In the experiment, the DBD plasma device, positioned approximately 10 mM above cells in a 96 or 48 healthy culture plate, facilitates the delivery of reactive species to the cell surfaces (Fig. 1b). Before plasma treatment, a portion of the cell culture medium was gently removed, leaving approximately 50 μL with a liquid depth of around 2 mM. In contrast, the cells remained adhered to the bottom. Following 1, 2, or 3 min of DBD plasma exposure, fresh medium was added to each dish, and the cells were cultured for 72 h before detection. The entire experiment was repeated three times to ensure accurate and reliable results (Fig. 7).

### Cell culturing

The HT29, CT26, LOVO, MC38, and HCT116 colorectal cancer cell lines were obtained from Professor Shu Zhu's Laboratory at the University of Science and Technology of China. Each cell line was cultured separately in DMEM medium (Biological Industries 04-001-1ACS) supplemented with 10% (v/v) fetal bovine serum (FBS, BI), 1% (v/v) penicillin-streptomycin (BI), 1 mM sodium pyruvate, and 2 mM l-glutamine at 37 °C with 5% CO2 in humidified conditions^57^. All experiments were conducted using cells at passages 4-6 to ensure consistency and reliability in the obtained results.

### Plasma paraments measurement

Gas-phase plasma determination is used for plasma diagnosis. Plasma characteristics are determined by measuring the current, voltage, charge, and emission spectrum. During DBD discharge device operation, the voltage at the end of the plasma generator was measured using the PINTECH HVP-15HF 1000:1 attenuation high-voltage probe. The discharge current was measured by the PINTECH PT710-D current sensor connected in a series in the discharge circuit of the reactor. The discharge power (P) of the CAP device can be calculated using the following formula:

$${\text{P}}=\frac{1}{{\text{T}}}{\oint }_{{\text{T}}}{\text{Q}}({\text{v}}){\text{dv}}$$

We further derive from the above equation:

$${\text{P}}={\text{f}}\times {\text{C}}\times {\text{s}}$$

The discharge cycle of the DBD plasma device is denoted by T, and the voltage by V. The discharge frequency is represented by f. At the same time, C (1.0 μF) is the sampling capacitance value, and s (375.2) is the area of the Lissajous figure (Fig. 1d). The data of the DBD discharge voltage and current waveform are presented in Fig. 1c. Based on our results, the present study was conducted at a current of 129.8 mA, voltage of 6.495 kV, frequency of 14.58 kHz, and power of 5.47 W. These parameters were carefully chosen to ensure effective and safe plasma treatment of colorectal cancer cells in vitro.

### Plasma qualitatively analysis

Optical emission spectroscopy (OES) primarily serves for the qualitative assessment of CAP's reactive oxides and nitrogen species, with substance identification relying on spectral peak positions ( Fig.s 8). Molecular transitions of the nitrogen second positive system (N_2_ SPS) are indicated within the range of 354–425 nm (354.40 nm, 358.32 nm, 371.65 nm, 376.23 nm and 381.04 nm)^58–61^.The nitrogen first negative system (N_2_ FNS) emissions are observed at wavelengths ranged 390–440 nm (392.01 nm, 394.75 nm, 400.45 nm, 406.59 nm, 420.43 nm, 427.45 nm and 434.67 nm)^58–60^. The second-order nitrogen molecules N_2_ are observed at wavelengths ranged 595–775 nm (595.99 nm, 627.75 nm, 632.41 nm, 674.8 nm, 707.88 nm, 715.46 nm, 751.42 and 761.47 nm)^62,63^.The emissions, arising from excited nitrogen species, could result from nitrogen molecules present in both the feeding gas and the ambient environment. There are also emissions from atomic nitrogen(Atomic N) (742.33 nm and 747.38 nm)^59,60^.There is an emission peak of atomic oxygen(Atomic O) manifests at 777.86 nm which is due to the dissociation of oxygen molecules^59,60,64^. The emission peak at 656.41 nm respects from hydrogen atom(Hα)^58^. This observation validates that the devices can generate reactive oxides and nitrogen species (ROS/RNS).

**Figure 8:**
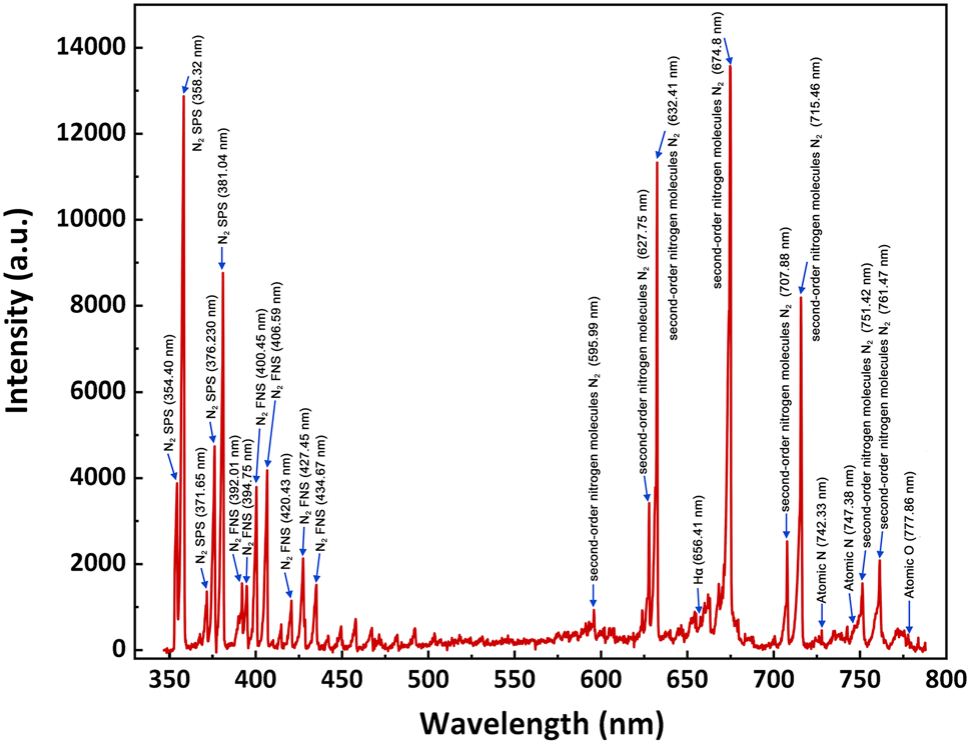
The optical emission spectroscopy of the DBD plasma device is in the wavelength range of 350–800 nm.

### Cell cytotoxicity assay

Colorectal cancer cells (HT29, CT26, LOVO, MC38, and HCT116) were seeded overnight in 96 well plates at a density of 2 × 10^5^ cells per well. The cells were treated with CAP for 60, 120, or 180 s and cultured for 72 h. Subsequently, cytotoxicity was assessed by measuring lactate dehydrogenase (LDH) release. Cells were incubated in 150 μL PBS with 10% LDH-releasing buffer (LDH Beyotime Biotechnology Cat# C0016) for 1 h, and absorbance was measured at 490 nm (OD490) after adding the LDH-detecting mix buffer.

### Cell death detection

Colorectal cancer cell lines (HT29, CT26, LOVO, MC38, and HCT116) were seeded in 48 well plates (5 × 10^5^ per well) CAP treatment was administered for 60, 120, and 180 s. Followed by a 72-h culture, cells were washed and suspended in 50 µL of PBS. Apoptosis analysis was conducted using Annexin V-FITC and PI (Propidium Iodide) staining assay kit (Cat# C1062L, Beyotime Biotechnology, Shanghai, China) for 15 min at room temperature in the dark. The stained cells were then analyzed using a flow cytometer (CytoFLEX, Beckman, Miami, FL, USA), and the resulting data were analyzed with the FlowJo software (Tree Star, Ashland, OR, USA). Colorectal cancer cells (HCT116, LOVO, MC38, HT29, and CT26 cells), following pretreatment with ROS scavenger (NAC) or NO scavenger Carboxy-PTIO, were also analyzed by flow cytometry after exposure to 180 s of CAP, as described above.

### Lipid peroxidation determination

HT29, CT26, LOVO, MC38, and HCT116 cells were seeded in 96 well plate (5 × 10^5^ per well) overnight before CAP treatment. After 72 h of culture, cells were lysed using Western and immunoprecipitation lysis buffer 50 μL per well (Cat# P0013, Beyotime Biotechnology, Shanghai, China). The lysates were homogenized, and the resulting homogenates were centrifuged at 1,600xg at 4 °C for 10 min. The supernatants were collected and assessed using the Lipid Peroxidation Malondialdehyde (MDA) Assay Kit (Cat# S0131, Beyotime Biotechnology, Shanghai, China). Specifically, 200 µL of thiobarbituric acid (TBA) reagent was added to 100 µL of the cell suspension, and the mixture was heated in a boiling water bath for 15 min. After cooling and centrifuging the reaction mixture at 1,000xg for 10 min, the supernatant was separated, and absorbance was measured at 530 nm using the multi-mode microplate reader (SYNERGY H1, BioTek, Vermont, VT, USA). MDA levels were expressed as units (U) per 1 × 10^6^/mL cells and as nmol/mg protein.

### Measurement of intracellular/extracellular ROS & RNS

Fluorescence probes DCFH-DA (Cat# S0033S, Beyotime Biotechnology, Shanghai, China), Dihydroethidium (DHE) (Cat# S0063, Beyotime Biotechnology, Shanghai, China) and DAF-FM DA (Cat# S0019, Beyotime Biotechnology, Shanghai, China) were employed to measure intracellular ROS and RNS in colorectal cancer cells. DCFH-DA is a fluorescent probe detecting intracellular total reactive oxygen species (ROS) levels. At the same time, DAF-FM DA monitors nitric oxide (NO) production in living cells. Dihydroethidium (DHE), another fluorescent probe, enters cells through the cell membrane. Intracellularly, it undergoes dehydrogenation influenced by the presence of intracellular superoxide anion, producing ethidium. Subsequently, ethidium binds to RNA or DNA, generating red fluorescence. The probes were used according to the manufacturer's instructions. The cells (2 × 10^5^ per well in 96 well plates) were exposed to CAP before being stained separately with DCFH-DA, DHE, and DAF-FM DA solutions for 1 h in the dark at room temperature. After washing the cells with PBS thrice, subsequently, fluorescence intensity was measured at specific excitation/emission wavelengths (488/525 nm for ROS, 300/610 nm for superoxide anion, and 495/515 nm for nitric oxide). The fluorescence intensity was detected by the multi-mode microplate reader (SYNERGY H1, BioTek, Vermont, VT, USA).

Extracellular ROS/RNS levels were determined by measuring H_2_O_2_ and Total Nitric Oxide concentrations in the culture medium using the H2O2 assay kit (Cat#S0038, Beyotime Biotechnology, Shanghai, China) and NO assay kit (Cat# S0023, Beyotime Biotechnology, Shanghai, China). Following the manufacturer's instructions, in a 96-well plate, 2 × 10^5^ cells per well were exposed to CAP treatment. Cells were removed, and the supernatants of tumor cell cultures were collected for H_2_O_2_ and NO assays. Due to the instability of NO, it is prone to rapid metabolism into nitrite (NO2^−^) and nitrate (NO3^−^), so we measured nitrite and nitrate concentration to assess NO levels.

N-acetyl-L-cysteine (NAC) is a ROS scavenger (CAT# S0077, Beyotime Biotechnology, Shanghai, China), and Carboxy-PTIO is an NO scavenger, (CAT# S1546, Beyotime Biotechnology, Shanghai, China). Colorectal cancer cells were pre-treated with the indicated concentrations of ROS scavenger (NAC) (5mM) or NO scavenger Carboxy-PTIO (50 µM) for 2 h before exposure to 180 s of CAP treatment. Subsequently, cells were collected, and LDH, ROS, and RNS levels were assessed as described above.

### Western blot

Western blotting was performed to evaluate alterations in the expression of cell apoptosis and MMR-associated proteins. Cells were seeded into 48 well plate (5 × 10^5^ per well) overnight before CAP treatment. Then, cells from both test and control groups were lysed in 100 μL RIPA buffer per well (Cat# P0013, Beyotime Biotechnology, Shanghai, China) and centrifuged at 12,000 rpm for 10 min at 4 °C to collect the supernatants. A 25 mg/mL protein standard was prepared by adjusting the concentration to be consistent with RIPA, mixing it evenly with the protein loading buffer, and boiling it for 10 min to denature the protein. The protein samples were loaded onto an SDS-PAGE gel (10 μL per well) and transferred to a PVDF membrane for electrophoresis. The membrane was blocked with non-fat dry milk for 1 h at room temperature and then incubated with specific primary antibodies, including Bcl-2 (Cat# 26593-1-AP, ProteinTech Chicago, IL, USA), Bax (Cat# 50599-2-Ig, ProteinTech Chicago, IL, USA), caspase 3 (Cat# 19677-1-AP, ProteinTech Chicago, IL, USA), caspase 9 (Cat# 10380-1-AP, ProteinTech Chicago, IL, USA), Cyt C (Cat# 10993-1-AP, ProteinTech Chicago, IL, USA), anti-MLH1 (Cat# 11697-1-AP, ProteinTech Chicago, IL, USA) and anti-MSH2 (Cat# 60161-1-Ig, ProteinTech Chicago, IL, USA) overnight at 4 °C. The information on all antibodies is shown in Table S2. Following washing with PBST three times for 15 min each, the membrane was immersed in the secondary antibody working solution (secondary antibodies: sealing solution =1:5,000) for 1 h at room temperature. After washing with PBST three times, the intensity of the scanned bands was analyzed with High-sig ECL Western Blotting Substrate kits (Cat# 180–501, Tanon, Shanghai, China), and the images were acquired using the Fujifilm Luminescent Image Analyzer LAS 4000. In Fig. 5, the purpose of WB here is to compare the expression of proteins within the same cell line rather than across different cell lines. To avoid some protein bands being overexposed while others are underexposed due to significant differences in optimal exposure time among different cell lines, membranes of varying cell line samples were cut and separated before development to obtain the optimal western blotting photos.

#### Quantitative RT-PCR analysis

Total RNA was isolated from the tumor cells using an RNA extraction kit (Cat# 9109, Takara, Tokyo, Japan). The isolated RNA (500 ng) was reverse transcribed into cDNA with the HiScript III RT SuperMix kit (Cat# R323–01, Vazyme, Piscataway, NJ, USA) following the manufacturer's instructions. PCR was carried out in a volume of 20 μL containing 12.5 μL 2 × PCR Mix (BioChain, San Francisco, CA, USA), 2 μL cDNA, 0.3 μL EvaGreen (Biotium, Bay area, CA, USA), 0.5 μL of primers each, and 4.2 μL DEPC-treated water. The mixtures were amplified by an initial denaturation step of 95 °C for 5 min, 45 cycles of denaturation at 95 °C for 30 s, annealing at 60 °C for 30 s, and extension at 72 °C for 30 s, and a final elongation step at 72 °C for 5 min. Quantitative PCR was performed using the ChamQ Universal SYBR qPCR Master Mix (Cat# Q711–02, Vazyme, Piscataway, NJ, USA) in a fluorescence quantitative PCR apparatus LightCycler96 System (Roche, Basel, Switzerland). The relative expression levels of target genes mRNA were calculated by the ΔΔCT-method relative to the internal standard Glyceraldehyde 3-phosphate dehydrogenase (GAPDH) and control lane (E2 = 0). The study employed three repetitions for each sample and used GAPDH as the reference gene. The PCR primer sequences for the target genes MLH1, MSH2, and GAPDH are listed in Table S1.

#### Statistical analysis

The data were obtained from three independent experiments and expressed as mean ± standard deviation (SD). Unpaired Student's t-test in the GraphPad Prism® software program (version 6, Graphpad Software, Inc.) was used for statistical analysis. A *p* value less than 0.05 was considered statistically significant. All experiments were repeated three times for the validity and reproducibility of the results.

### Supplementary Information

Supplementary Information 1.

Supplementary Figures.

## Data Availability

The datasets generated and/or analyzed during the current study are available in the [WB Blotting.zip] repository. [https://submission.springernature.com/submission/8d3029cb-4be8-4196-b0a0-2ae61c7cc2b0/file/94b5120a-a547-44f0-9e46-fd330e9947d8].
