# Supplementary material for: Cold atmospheric plasma stabilizes mismatch repair for effective, uniform treatment of diverse colorectal cancer cell types
Source: Sci Rep. 2024 Feb 13;14:3599. doi: 10.1038/s41598-024-54020-0 (PMC10864286; doi:10.1038/s41598-024-54020-0)
Supplement: Supplementary file 1 — Supplementary Information 1. [file 41598_2024_54020_MOESM1_ESM.docx]

Supplementary Information

**Cold Atmospheric Plasma Stabilizes Mismatch Repair for Effective, Uniform Treatment of Diverse Colorectal Cancer Cell Types**

**Yuanyuan He^1,2#^, Fu Lu^1^, Chenmin Jiang^3^, Fanwu Gong^4*^, Zhengwei Wu^1 *^and Kostya Ostrikov^5^**

^1^ School of Nuclear Science and Technology, University of Science and Technology of China, Hefei, 230026, China

^2^ Department of Geriatrics, The First Affiliated Hospital of USTC, Division of Life Sciences and Medicine, University of Science and Technology of China, Hefei 230001, China

^3^ School of Pharmacy, Anhui Medical University, Hefei, Anhui 230032, China

^4^ Department of Medical Oncology, The First Affiliated Hospital of USTC, Division of Life Sciences and Medicine, University of Science and Technology of China, Hefei 230001, China

^5^ School of Chemistry and Physics and QUT Centre for Biomedical Technologies, Queensland University of Technology (QUT), Brisbane, Queensland 4000, Australia

* Correspondence: [wuzw@ustc.edu.cn](mailto:wuzw@ustc.edu.cn) (Z.W. Wu) and gongfw@mail.ustc.edu.cn (F.W. Gong);


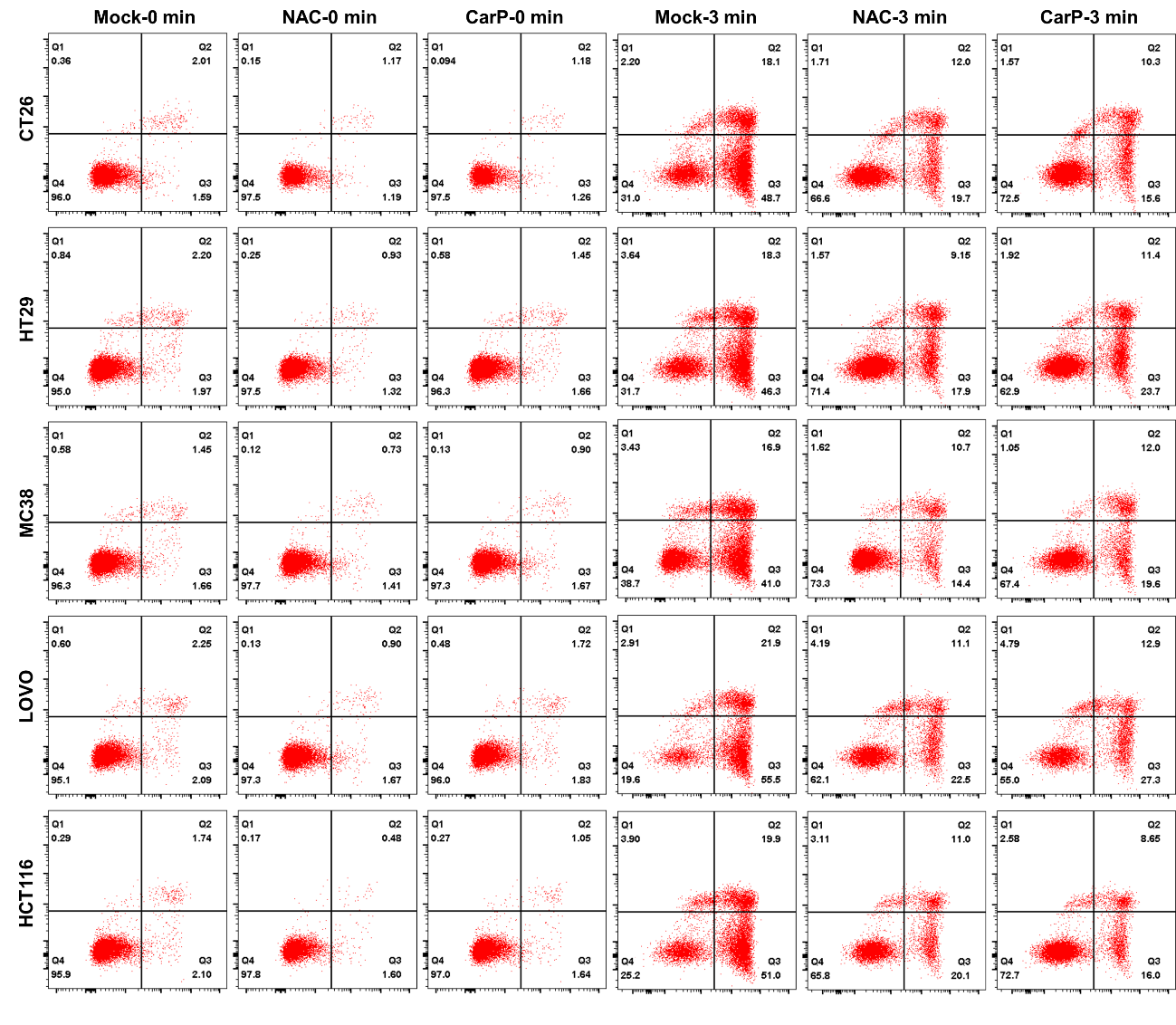


Figure S1. N-acetyl-L-cysteine (NAC) and Carboxy-PTIO inhibited CAP-induced apoptosis in tumor cells.

Flow cytometry logarithmic plots of apoptotic cells in all groups (control, NAC treatment and Carboxy-PTIO treatment) are described in Figure 3, n = 6.

Table S1. Primers for q-PCR analysis of mouse (M) and human (H) derived samples in this study.

Table S2. Antibodies for western blotting of indicated proteins in this study.
