# Supplementary material for: Cold atmospheric plasma stabilizes mismatch repair for effective, uniform treatment of diverse colorectal cancer cell types
Source: Sci Rep. 2024 Feb 13;14:3599. doi: 10.1038/s41598-024-54020-0 (PMC10864286; doi:10.1038/s41598-024-54020-0)

Manuscript Fig 5a

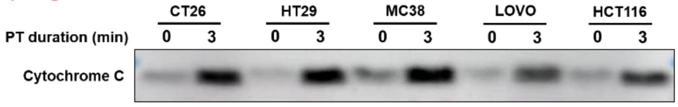

Original image

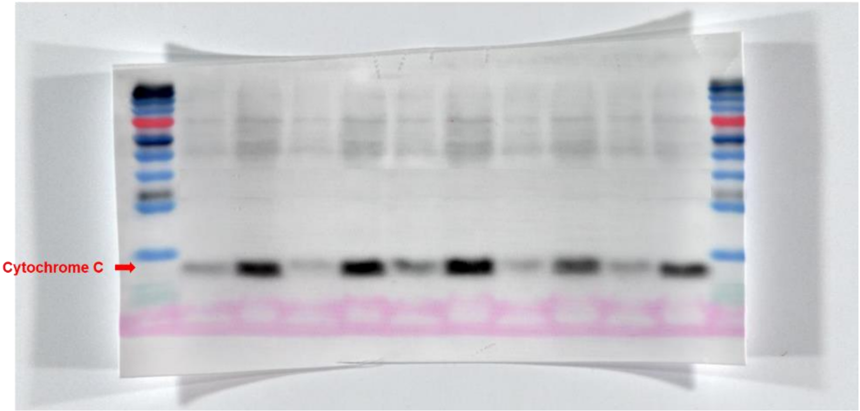

Manuscript Fig 5a

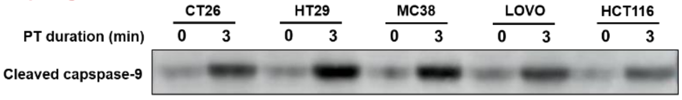

Original image

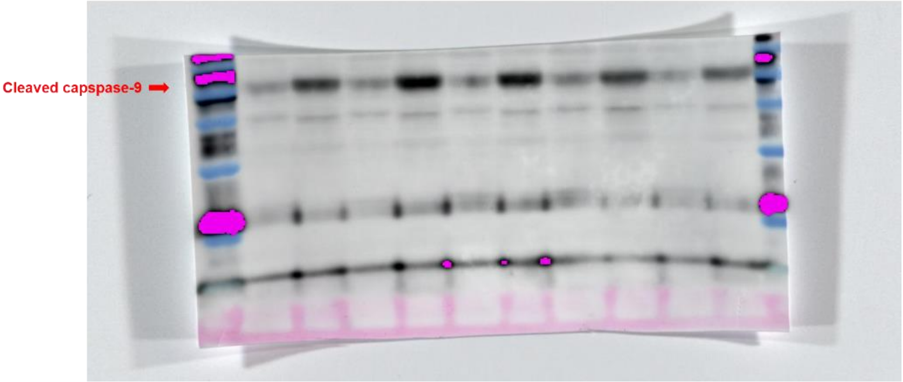

Manuscript Fig 5a

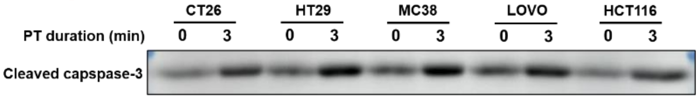

Original image

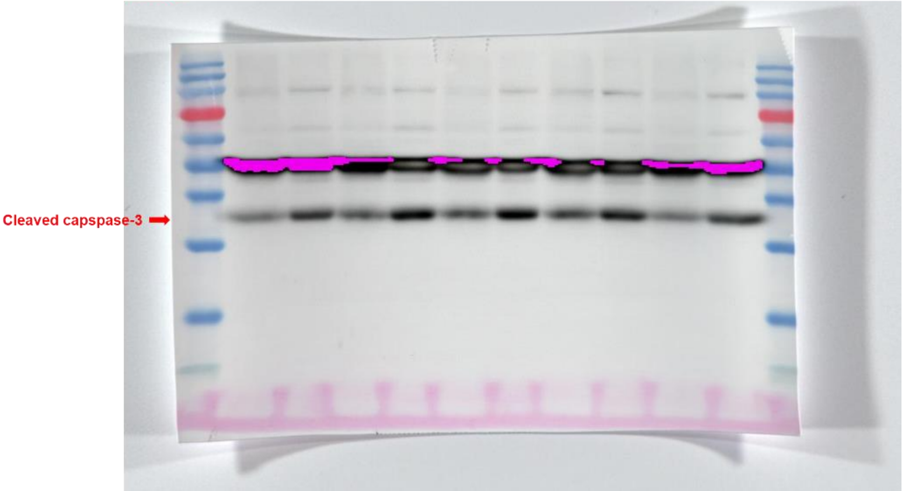

Manuscript Fig 5a

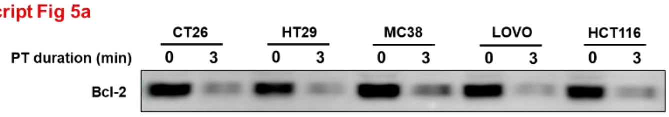

Original image

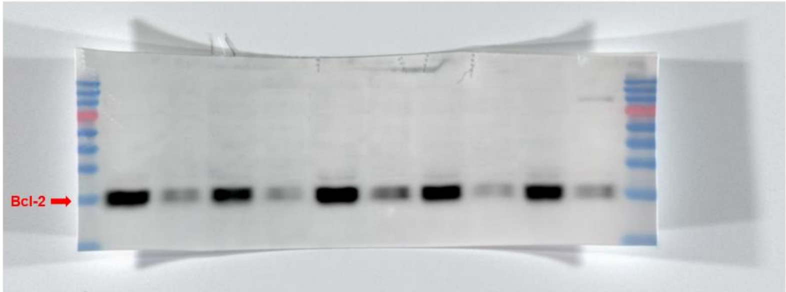

Manuscript Fig 5a

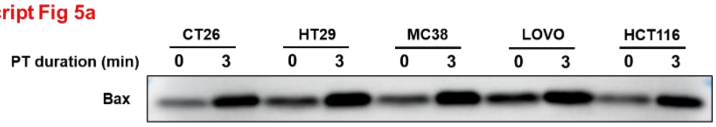

Original image

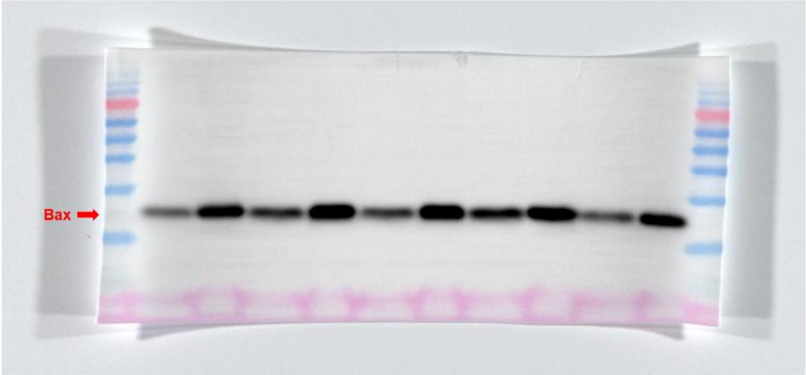

Manuscript Fig 5a

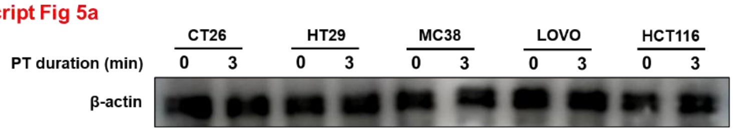

Original image

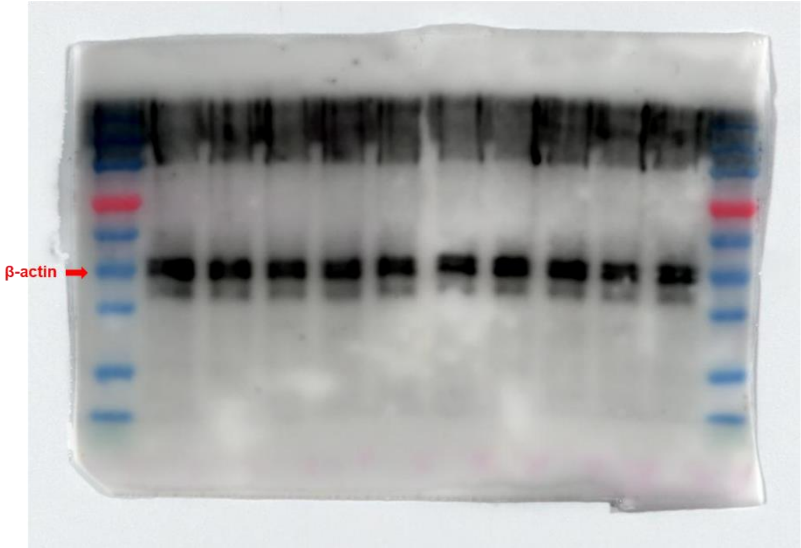

Manuscript Fig 6a

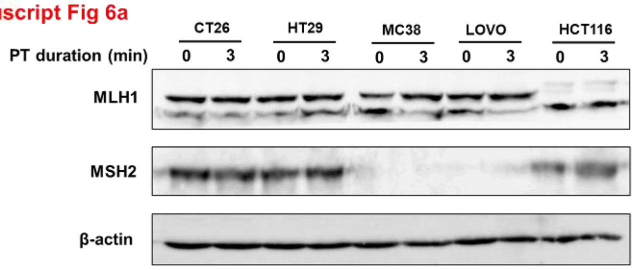

Original image

MLH1

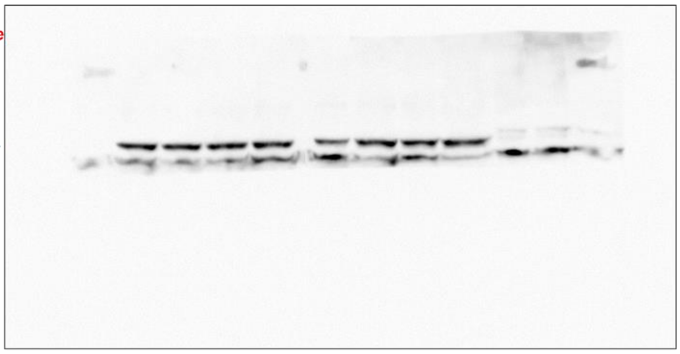

MSH2

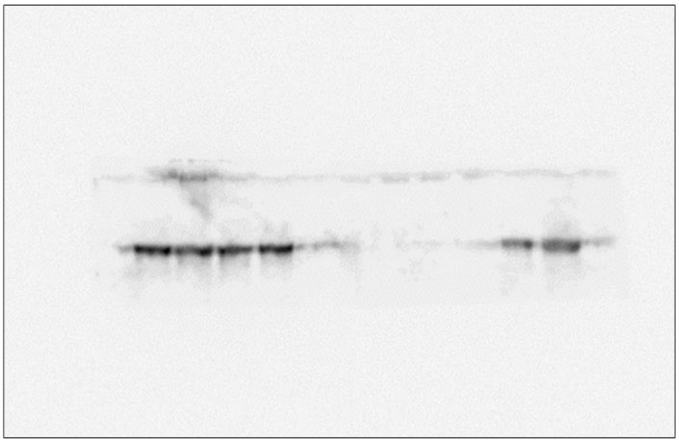

β-actin

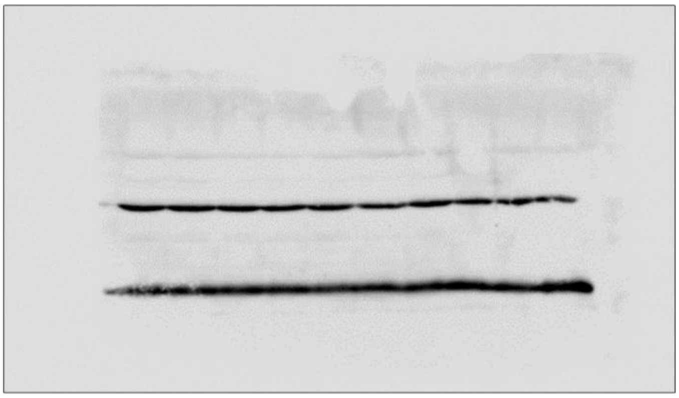

Marker

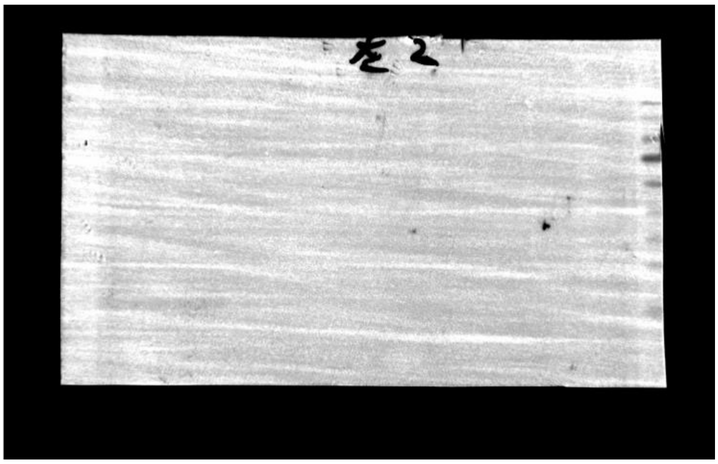

Supplement: Supplementary file 2 — Supplementary Figures. [file 41598_2024_54020_MOESM2_ESM.pdf]
